# Canonical Wnt signalling from the area opaca induces and maintains the marginal zone in pre-primitive-streak stage chick embryos

**DOI:** 10.1242/dev.204350

**Published:** 2025-01-23

**Authors:** Yara Fadaili, Hui-Chun Lu, Hyung Chul Lee, Amra Ryazapova, Claudio D. Stern

**Affiliations:** Department of Cell and Developmental Biology, University College London, Gower Street, London WC1E 6BT, UK

**Keywords:** Nieuwkoop centre, Extra-embryonic tissues, Organizer, Gastrulation, Primitive streak, Amniote embryos, Chick

## Abstract

In chick embryos before primitive streak formation, the outermost extra-embryonic region, known as the area opaca (AO), was generally thought to act only by providing nutrients and mechanical support to the embryo. Immediately internal to the AO is a ring of epiblast called the marginal zone (MZ), separating the former from the inner area pellucida (AP) epiblast. The MZ does not contribute cells to any part of the embryo but is involved in determining the position of primitive streak formation from the adjacent AP epiblast. Recently, it was discovered that the AO can induce an MZ from AP epiblast. Here, we explore the nature of this inductive signal. We find that *WNT8C* is highly expressed in the AO, whereas canonical Wnt pathway targets are enriched in the MZ, along with strong nuclear β-catenin localization. Using isolation and recombination experiments combined with gain- and loss-of-function by exogenous chemical modulators of the pathway, we reveal that Wnt signalling is essential for induction and maintenance of the MZ, as well as sufficient to induce MZ properties in AP epiblast. We propose that canonical Wnt signalling is responsible for induction of the MZ by the area opaca.

## Teaser

**Summary:** Canonical Wnt is the signal from the extra-embryonic area opaca that induces and maintains the neighbouring marginal zone of the chick embryo, crucial for positioning the primitive streak.

## INTRODUCTION

**Fig. 1.**
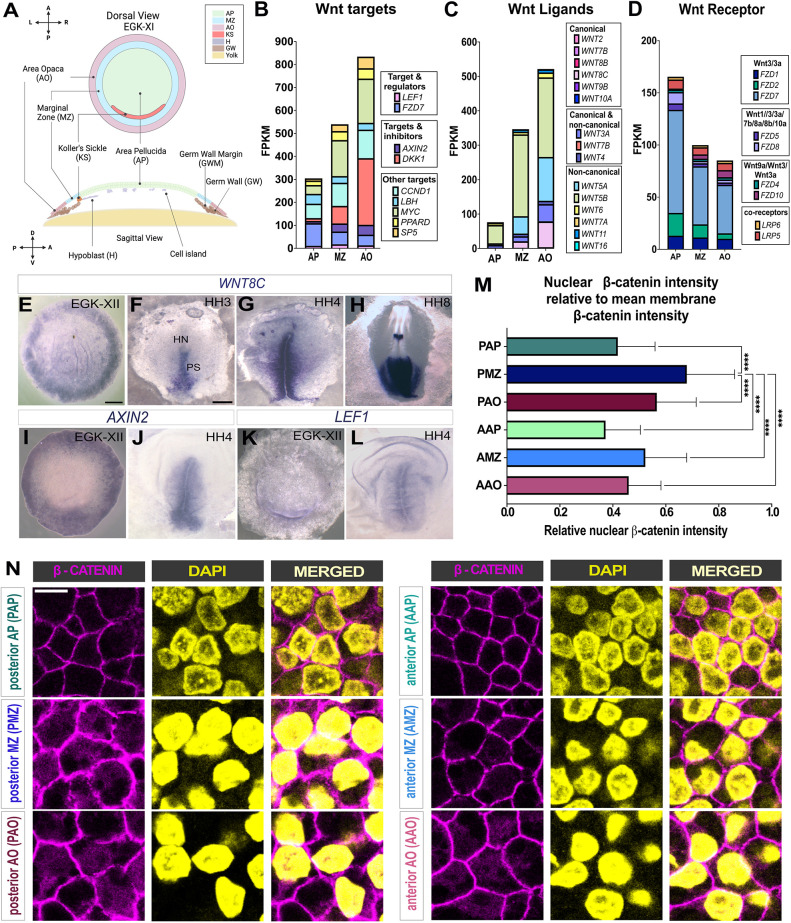
**Spatial and regional expression of canonical Wnt ligands, targets and effectors.** (A) Schematic of the different regions of a stage EGK-XI embryo. The yellow area beneath the embryo in the sagittal view represents the yolk. Created in BioRender. Fadaili, Y. (2025) https://BioRender.com/d35p395. (B-D) Stacked bar chart of RNA-sequencing data analysis of different regions collected from stage EGK-XII embryos (Lee et al., 2020). Whole AO, whole MZ and whole AP were analysed to compare FPKM values of Wnt-related molecules, grouped into three categories: Wnt targets (B), Wnt ligands (C) and Wnt receptors (D). (E-H) Whole mount *in situ* hybridization of canonical Wnt ligand *WNT8C* at different stages: EGK-XII (E), HH3 (F), HH4 (G) and HH8 (H). (I-L) *In situ* hybridization of Wnt targets *AXIN2* (I,J) and *LEF-1* (K,L) in embryos at stages EGK-XI (I,K) and HH4 (K,L). The embryos are viewed from the dorsal (epiblast) side. (M,N) Regional differences in β-catenin localization in early pre-gastrulation chicken embryo. (M) Bar graph representing nuclear β-catenin pixel intensity (obtained from nuclear segmentation of DAPI) relative to the mean membrane intensity (obtained by membrane β-catenin segmentation) (*n*=3 separate embryos; Kruskal–Wallis *t*-test; ****P<0.0001) between all regions compared to the PMZ. Data are mean±s.e.m. (N) β-catenin staining (maximum projection from a stack of ten *z*-slices). AAO, anterior area opaca; PAO, posterior area opaca; AMZ, anterior marginal zone; PMZ, posterior marginal zone; AAP, anterior area pellucida; PAP, posterior area pellucida. Scale bars: 1mm (E-L); 10 μm (N).

The chick embryo at stage X, when the egg is laid, is a flat disc-shaped blastoderm with a continuous epiblast layer that is divided into three concentric regions (Fig. 1A). The central region, known as the area pellucida (AP), contains all cells that will contribute to the embryo (Eyal-Giladi and Kochav, 1976; Hatada and Stern, 1994; Lee et al., 2020). A ring of epiblast immediately surrounding the AP (delimited from it posteriorly by Koller's sickle) is called the marginal zone (MZ). Its cells do not contribute to any embryonic tissues, but it is involved in the establishment and maintenance of embryonic polarity, specifically by determining the location at the posterior edge of the AP, where primitive streak formation will begin (Eyal-Giladi and Kochav, 1976; Eyal-Giladi and Khaner, 1989; Bachvarova et al., 1998; Torlopp et al., 2014; Lee et al., 2020, 2024b). The outermost region, called the area opaca (AO), which is also extra-embryonic, like the MZ, has generally been considered to provide nutrients and mechanical support for blastoderm expansion, but otherwise to have no instructive role in morphogenesis of the embryo (Bellairs et al., 1967; Downie, 1976; Lee et al., 2022a, 2024a).

A new role for the AO has recently been uncovered. When the MZ is removed and the AO made to surround the central AP directly, a new MZ is induced, expressing specific gene markers such as *ASTL* (Lee et al., 2022b). This finding suggests that the AO plays a role in establishment of the MZ and thereby positioning of the primitive streak. The question remains as to the nature of the signalling molecules emitted by the AO that are responsible for the induction of MZ character.

Here, we address this question. First, we survey expression of secreted factors and their corresponding receptors in these three different regions of the early embryo using CellChatDB (Jin et al., 2021) ligand-receptor annotations to the RNA-seq data and by *in situ* hybridization. This initial survey points to canonical Wnt as a possible candidate. Indeed, we find that targets of this pathway are highly expressed in the MZ, as is nuclear localization of β-catenin, indicating that this region receives strong Wnt signals in the normal embryo. Next, we explored the role of canonical Wnt in the AO more directly by loss-of-function using the Tankyrase inhibitor IWR-1, and gain-of-function with the GSK-3 inhibitor BIO. Wnt inhibition hindered the ability of the AO to induce a new MZ from AP epiblast, whereas BIO increased the inductive ability of the AO. When an isolated AP is treated with BIO, it loses AP markers and gains expression of MZ markers, suggesting that Wnt is sufficient to transform the entire AP into a MZ. Moreover, normal, intact stage X embryos, which possess an established MZ, lose MZ markers when treated with IWR-1, suggesting that continued Wnt signalling is required to maintain the MZ after its establishment in normal development. We therefore propose that canonical Wnt signalling is the MZ-inducing signal from the AO. This is reminiscent of the role of canonical Wnt signalling in dorsoventral patterning and for establishing the Nieuwkoop centre in *Xenopus* embryos (Funayama et al., 1995; Guger and Gumbiner, 1995; He et al., 1995; Schneider et al., 1996; Yost et al., 1996, 1998).

## RESULTS

### High Wnt signalling activity in the area opaca and marginal zone of the early chick embryo

To identify candidate signalling pathways that are active in the AO and can be received by the responding AP, we started by exploring which ligands are expressed in the AO (as the signalling region) that have corresponding receptors expressed in either the AP (which is competent to respond to signals from the AO) or the MZ (which has presumably been induced by these signals earlier). We used the CellChatDB ligand-receptor annotations (http://www.cellchat.org/cellchatdb/) (Jin et al., 2021) to analyze gene expression data in fragments per kilobase of transcript per million mapped reads (FPKM) obtained from RNA-seq data (Lee et al., 2020) for stage EGK XII embryos (Eyal-Giladi and Kochav, 1976), for the whole area opaca (wAO), whole area pellucida (wAP) and whole marginal zone (wMZ). The results are presented in Table S1. Some prominent candidates include Wnt ligands (especially WNT3A, 5A, 5B and 8C) and their Fz receptors, Apela/Aplnr, BMPs and Nodal. The canonical Wnt co-receptors *LRP5*/*6* are also expressed in the responding tissue (Table S1).

To explore this further, we compared the expression of individual Wnt targets (Fig. 1B), ligands (Fig. 1C) and receptors (Fig. 1D) in these regions. The average FPKM values of all canonical Wnt targets is 2.7-fold higher in the AO and 1.8-fold higher in the MZ compared to the AP (Fig. 1B). The FPKM value of Wnt ligands is 1.5-fold higher in the AO than in the MZ and 6.8-fold higher than in the AP (Fig. 1C). Wnt receptors, on the other hand, show a complementary expression profile, with the combined FPKM values of the receptors in the AP being 1.6-fold higher than in AO and 1.2-fold higher in the MZ than in the AO (Fig. 1D).

Among the canonical Wnt ligands, *WNT8C* is expressed in the AO at a 4-fold higher level than in the MZ, and no significant expression is detected in the AP (Fig. 1C). This expression pattern was confirmed by *WNT8C in situ* hybridization of pre-primitive-streak stage embryos (Fig. 1E) and agrees with earlier findings (Hume and Dodd, 1993; Skromne and Stern, 2002). Similarly, the Wnt target and modulator *AXIN2* reveals overlapping expression with *WNT8C. AXIN2* has higher expression in the AO and MZ, but no expression is detected in the AP (Fig. 1I). The expression of another Wnt target, *LEF-1*, is confined to the posterior MZ in EGK-XI embryos (Fig. 1K). Together, these expression patterns suggest that canonical Wnt signals, probably conveyed by WNT8C, are produced mainly by the extra-embryonic AO and received by the MZ. The high expression of Wnt receptors but not Wnt targets in the AP is consistent with this region being competent to respond to Wnt signals, but also suggests that at this stage the signal does not extend significantly beyond the MZ. We also observed that once the primitive streak forms at stage HH-2 (Hamburger and Hamilton, 1951), *WNT8C* (Fig. 1F-H), *AXIN2* (Fig. 1J) and *LEF-1* (Fig. 1L) expression are all cleared from the AO and MZ and instead become expressed in the primitive streak itself, suggesting a different role of Wnt signalling at this later stage. Moreover, since the AO loses its ability to induce MZ character at stage HH-2 (Lee et al., 2022b), the expression of *WNT8C* and its targets closely mirrors the stages at which the AO possesses this inductive property.

To confirm these findings, we performed immunostaining for the canonical Wnt effector β-catenin. We quantified nuclear β-catenin staining intensity relative to the membrane intensity in segmented images of six different regions: posterior-AO, -MZ and -AP, and anterior-AO, -MZ and -AP. The highest nuclear intensity of β-catenin was seen in the posterior side of the embryo, and the AO and MZ (both anterior and posterior sides) showed higher nuclear intensity of β-catenin than the AP. The posterior MZ displayed the highest nuclear intensity of β-catenin, compared to all other embryonic and extra-embryonic regions (*P*-value <0.0001; Kruskal–Wallis test) (Roeser et al., 1999) (Fig. 1M,N).

Together, these results show that canonical Wnt signalling activity in pre-primitive streak stage embryos is concentrated in extra-embryonic regions, consistent with the expression of *WNT8C* and β-catenin described in previous studies (Roeser et al., 1999; Skromne and Stern, 2002; Schmidt et al., 2004; Lee et al., 2020). The expression profiles of Wnt ligands, receptors and targets therefore implicate Wnt signalling as a good candidate for the recently proposed signal from the AO that can induce MZ from responding AP cells (Lee et al., 2022b).

### Canonical Wnt signalling from the area opaca induces marginal zone identity in area pellucida epiblast

**Fig. 2.**
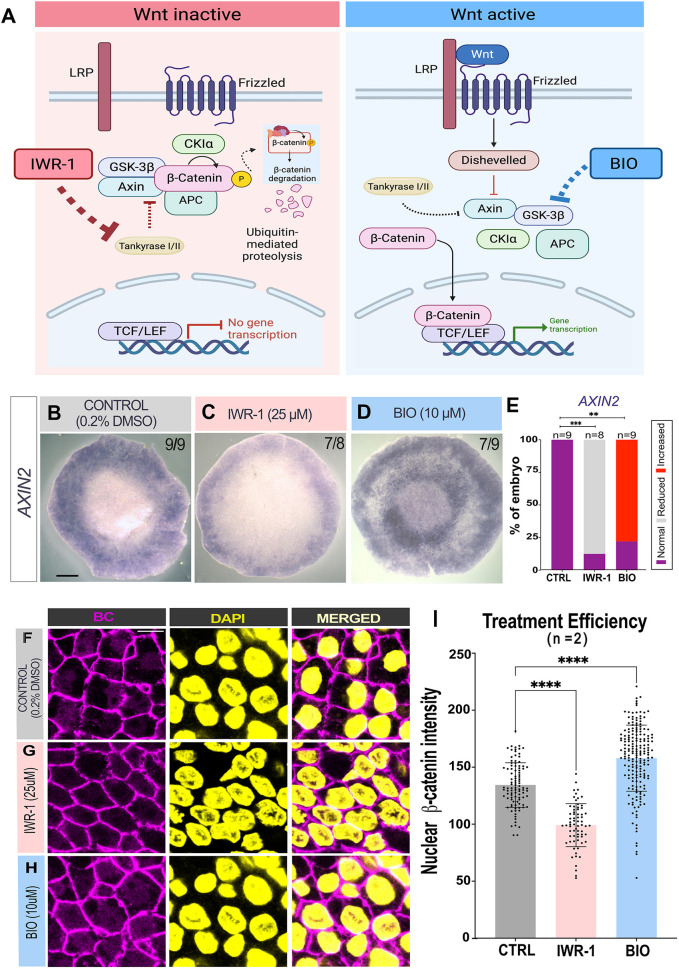
**Wnt signalling modulation by Wnt agonist BIO and antagonist IWR-1.** (A) Schematic of ‘active’ and ‘inactive’ Wnt signalling, highlighting the presence of the degradation complex in the absence of the Wnt ligand, which ultimately degrades β-catenin. When Wnt is present β-catenin is stabilized and can translocate to the nucleus. The sites of action of BIO (as a GSK inhibitor, Wnt agonist) and IWR-1 (Tankyrase inhibitor, Wnt antagonist) are shown. Created in BioRender. Fadaili, Y. (2025) https://BioRender.com/c10f689. (B-D) *In situ* hybridization of canonical Wnt target *AXIN2* after exposure to treatment for 7 h. Control (0.2% DMSO; B), IWR-1 (C) and BIO (D). (E) Stacked bar graph showing percentage of embryos with reduced, increased or normal expression. In the control sample (*n*=9), five embryos are stage EGK-XII and four embryos are stage HH2. IWR-1 (25 μM) *n*=8. *P*-value was determined using Fisher's exact test: IWR-1 versus control, ***P=0.0004; BIO versus control, **P=0.0023. (F-H) Confocal images of Fluorescent-IHC of β-catenin in the PAO of a *z*-stack maximum projection of EGK_XII treated embryos and control. DAPI, yellow; β-catenin, magenta. (I) Scatter bar plot showing the nuclear pixel intensity of β-catenin after 6 h of incubation of stage EGK-XI embryos incubated with: 0.2% DMSO for the control, IWR-1 (25 μM) and BIO (10 μM) (*n*=2). Each dot represents a segmented nucleus. Data are mean±s.e.m. Mann–Whitney *U*-test, ****P<0.0001. *n*, number of embryos. Scale bars: 1 mm (B-D); 5 μm (F-H).

The above findings suggest that Wnt signalling from the AO may be responsible for the induction of a new MZ from AP epiblast, after removal of the endogenous MZ. To test this, we performed ablation and recombination experiments combined with modulation of Wnt signalling. Wnt activity can be inhibited with the Wnt antagonist IWR-1 (25 μM), which inhibits Tankyrase (Narwal et al., 2012; Martins-Neves et al., 2018), or stimulated with the Wnt agonist BIO (10 μM), which inhibits GSK-3 (Meijer et al., 2003) (Fig. 2A). First, we validated the efficiency of these treatments. We incubated EGK-XI embryos with the Wnt modulators for 6 h and examined the expression of the Wnt target *AXIN2*, in comparison to embryos treated with 0.2% DMSO vehicle alone*.* Treatment with IWR-1 led to significant reduction in *AXIN2* expression in 7/8 embryos, a significant effect compared to embryos treated in vehicle alone (0.2% DMSO; 9/9 with normal expression) (Fisher's exact test, *P*=0.0004) (Fig. 2B-E). Conversely, treatment with BIO caused a significant increase in *AXIN2* expression (7/9 embryos) (Fisher's exact test, *P*=0.0023) (Fig. S1B-E). The effect of the pharmacological agents was also evaluated by immunostaining for β-catenin in the posterior AO. IWR-1 treated embryos showed significantly lower nuclear intensity of β-catenin in the posterior AO compared to 0.2% DMSO incubated controls (Mann–Whitney *U*-test, *P*<0.0001; *n*=2) (Fig. 2F,G,I), whereas embryos treated with BIO showed significantly higher nuclear intensity of β-catenin relative to controls (Fig. 2F,H,I) (Mann–Whitney *U*-test, *P*<0.0001; *n*=2). These results confirm that BIO and IWR-1 at these concentrations are effective as modulators of Wnt activity.

**Fig. 3.**
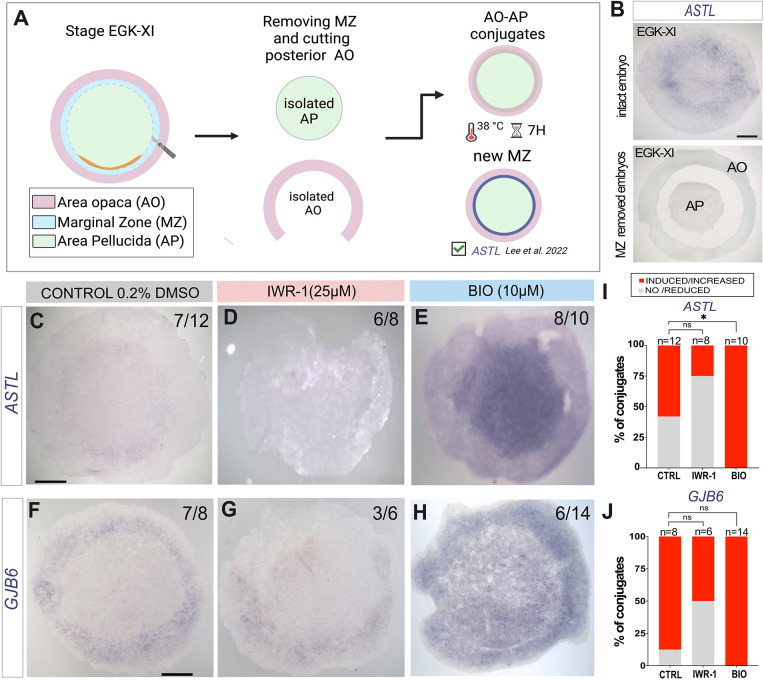
**Specification of the MZ and Wnt signalling.** (A) Schematic showing experimental design and possible results when removing the MZ and stitching the extra-embryonic AO onto the embryonic AP. Created in BioRender. Fadaili, Y. (2025) https://BioRender.com/a75b079. (B) Test for accuracy of ablation: *In situ* hybridization of MZ marker *ASTL* in intact and MZ ablated (AO+AP explants) EGK-XI embryos. (C-H) *In situ* hybridization of MZ markers ASTL (C-E) and GJB6 (F-H) in control AO-AP conjugates (0.2% DMSO; C,F), and conjugates treated with IWR-1 (25 μM; D,G) or BIO (10 μM; E,H) after 7 h incubation. (I,J) Stacked bar graphs representing percentage of AP-AO conjugates with induced or not induced ASTL (I) and GJB6 (J). *P*-values were determined by Fisher's test. *ASTL*: CTRL versus IWR-1 (*P*=0.1968; ns), CTRL versus BIO (*P=0.0396); *GJB6*: CTRL versus IWR-1 (*P*=0.0699; ns), CTRL versus BIO (*P*>0.999; ns). ns, not significant; CTRL, control. Scale bars: 1 mm.

With these tools, we assessed whether canonical Wnt signalling is required for the AO to induce MZ in AP epiblast (Lee et al., 2022b). The entire circumference of the MZ was excised from stage EGK-X embryos and the AO (with its posterior portion removed) juxtaposed onto the AP to surround it completely, to generate AO-AP conjugates (Fig. 3A), as previously described (Lee et al., 2022b). To check that the MZ had been completely removed, we performed *in situ* hybridization for the MZ marker *ASTL* in EGK-XI embryos immediately after ablation: indeed, no expression remained either in the isolated AO (0/2) or in the AP (0/8) (Fig. 3B). Induction of a new MZ was assessed after incubation of the AO-AP conjugates for 7 h by *in situ* hybridization for the MZ-specific genes *ASTL* and *GJB6*. We asked whether Wnt activity is necessary for MZ induction by the AO. In control (0.2% DMSO) incubated AO-AP conjugates, a new MZ was induced in the majority of cases, marked by expression of *ASTL* (7/12 conjugates) (Fig. 3C,I) and *GJB6* (7/8 conjugates) (Fig. 3F,I). When AO-AP conjugates were incubated with the Wnt antagonist IWR-1, the induction of MZ by the AO was reduced: 6/8 conjugates lacked *ASTL* expression (Fig. 3D,I) and 3/6 had no *GJB6* expression (Fig. 3G,J). These results suggest that Wnt signalling is required for the AO to induce a new MZ in the AP.

Next, we explored the effect of increased Wnt activity on this inductive interaction by treating AO-AP conjugates with BIO. This resulted in induction of a broader region of MZ than in controls, as revealed by *ASTL* (8/10 conjugates) (Fig. 3C,E,I) and *GJB6* expression (6/14) (Fig. 3F,H,J).

### Wnt signalling is sufficient to induce marginal zone identity in the area pellucida

**Fig. 4.**
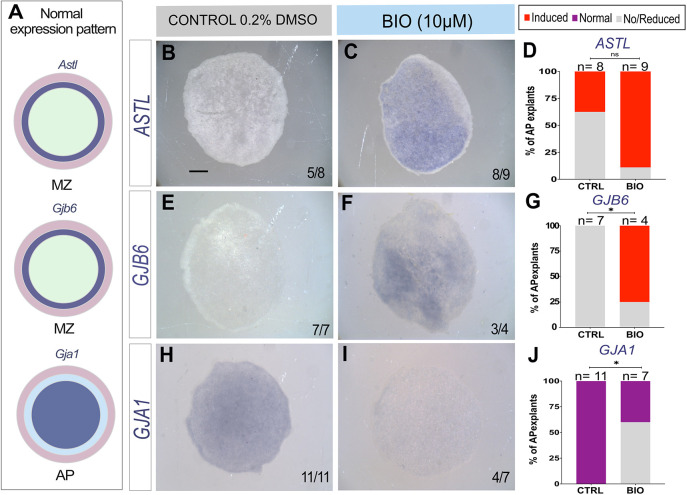
**Wnt stimulation induces MZ in AP explants.** (A) Summary of the normal expression domains of *ASTL*, *GJB6* and *GJA1* (expressing regions shown in dark blue). (B-J) *In situ* hybridization in control (0.2% DMSO; B,E,H) and Wnt-stimulated BIO-treated (10 μM, C,F,I) AP explants after 7 h culture; *ASTL* (B,C) *GJB6* (E,F) and *GJA1* (H,I). (D,G,J) Stacked bar graphs showing the percentage of AP explants with induced expression or no expression: *ASTL* (D), *GJB6* (G) and *GJA1* (J). *P*-values determined by Fisher's test: ASTL CTRL versus BIO (*P*=0.3168; ns), GJB6 CTRL versus BIO (*P=0.0242) and GJA1 CTRL versus BIO (*P=0.0114). Scale bar: 1 mm.

Although the results presented so far implicate Wnt in MZ induction, they do not rule out the possibility that another signal from the AO might be required along with Wnt in this process. To investigate whether Wnt activity alone is sufficient to induce MZ properties, we removed both the MZ and the AO and incubated the isolated AP in BIO for 7 h before assessing the expression of MZ gene markers *ASTL* and *GJB6* and of the AP marker *GJA1* (Fig. 4A) by *in situ* hybridization. *ASTL* expression was induced ectopically and very broadly in the AP after Wnt stimulation by BIO in 8/9 AP explants (Fig. 4C,D), whereas control (0.2% DMSO incubated) AP explants either showed no (5/8) or greatly reduced (3/8) expression (Fig. 4B,D). Moreover, expression of the AP-specific gap junction component *GJA1* was inhibited in the BIO-treated explants (4/7) (Fig. 4I,J), while the MZ specific gap junction *GJB6* was upregulated (3/4) (Fig. 4F,G). In contrast, control (0.2% DMSO) AP explants expressed *GJA1* (11/11; Fig. 4H,J) but not *GJB6* (4/4; Fig. 4E,G). These results suggest that Wnt is indeed sufficient to induce MZ identity in AP epiblast, even in the absence of the AO.

### The marginal zone is continuously and actively maintained by Wnt signalling

Is the continued presence of the AO required to maintain MZ properties in this region? To test this, we ablated the AO from EGK-XI embryos and incubated the AP+MZ explants for 7 h (Fig. S1A). *In situ* hybridization revealed that all AP+MZ explants (9/9) lost *ASTL* expression (Fig. S1C) compared to unoperated control embryos cultured for the same period (10/10) (Fig. S1B). This indicates that signals from the AO are required to maintain MZ properties during normal development, after its initial formation.

**Fig. 5.**
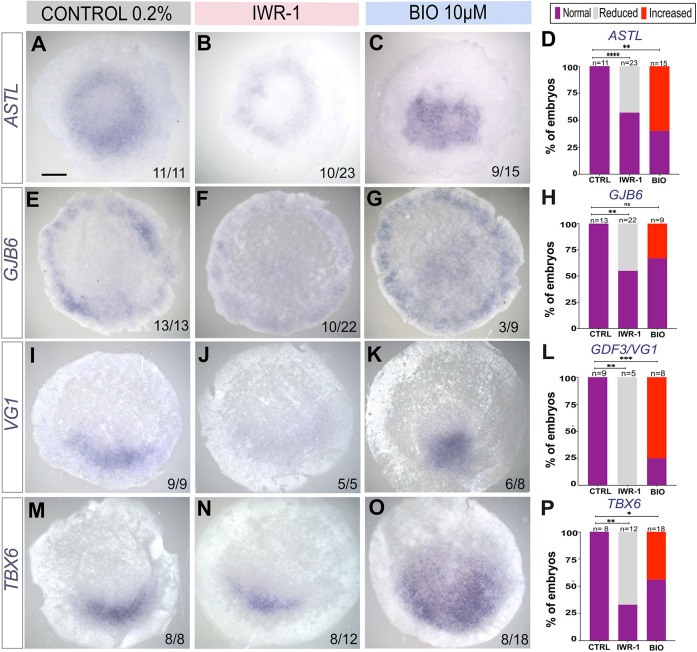
**Wnt signalling regulates MZ identity and posterior character.** (A-O) *In situ* hybridization showing expression of MZ-related genes *ASTL* (A-C) and *GJB6* (E-G) and the posterior MZ markers *VG1* (I-K) and *TBX6* (M-O) in control (0.2% DMSO; A,E,I,M), IWR-1 (25 μM) (B,F,J,N) and BIO (10 μM) (C,G,K,O) treated EGK-XI embryos incubated for 6 h. (D,H,L,P) Stacked bar graphs showing percentage of embryos with normal, reduced or increased expression compared to control of *ASTL* (D), *GJB6* (H), *VG1* (L) and *TBX6* (P). *P*-values were calculated by *Fisher's* exact (two sided) test; ns, not significant: *P*>0.05; *P<0.05; **P<0.005; ***P<0.0005; ****P<0.0001. Scale bar: 1 mm.

To test whether Wnt signalling is responsible for maintenance of the MZ, we incubated whole, unoperated stage XI embryos in the Wnt antagonist IWR-1 for 6 h and analysed the embryos by *in situ* hybridization. Expression of both *ASTL* and *GJB6* were inhibited: *ASTL* was reduced in 10/23 embryos (Fisher's exact test, *P*<0.0001), and *GJB6* was reduced in 10/22 embryos (Fisher's exact test, *P*=0.0052), compared to DMSO controls (Fig. 5A,B,E,D-F,H). We also examined the expression of the posterior MZ-expressed genes *VG1* (GDF3) and *TBX6*: 5/5 IWR-1-treated embryos showed reduced expression of *VG1* (Fig. 5J) (Fisher's exact test, *P*=0.0005) and 8/12 embryos showed reduced expression of *TBX6* (Fig. 5N) (Fisher's exact test, *P*=0.0047) compared to the 0.2% DMSO control embryos, where 9/9 embryos had normal expression of *VG1* and 8/8 embryos were normal for *TBX6* expression (Fig. 5I,M).

Treatment of whole EGK-XI embryos with BIO expanded the MZ into the AP, with broader *ASTL* (9/15 embryos) compared to the 0.2% DMSO incubated embryo, where 11/11 embryos showed normal expression (Fisher's exact test, *P*<0.0024) (Fig. 5C). However, a lower proportion (3/9) of treated embryos had expansion of the *GJB6* expression domain (Fisher's exact test, *P*<0.0545) (Fig. 5G). Wnt stimulation also led to expansion of the posterior MZ into the AP, revealed by expansion of *VG1* (6/8 embryos) (Fisher's exact test, *P*<0.0023) and *TBX6* (8/18 embryos) (Fisher's exact test, *P*<0.038) compared to controls (Fig. 5K,L,O,P). These findings suggest that Wnt activity is required for the maintenance of the MZ after its formation.

## DISCUSSION

This study reveals that canonical Wnt-signalling can account for the recently reported induction of MZ properties by the AO (Lee et al., 2022b). Inhibition of canonical Wnt signalling using IWR-1 prevents the AO from inducing MZ markers when recombined with the AP. Moreover, exposure of an isolated AP to the Wnt agonist BIO is sufficient to induce widespread expression of MZ markers. Furthermore, two findings suggest that continued signalling by the AO is required to maintain the MZ: first, ablation of the AO followed by culture of the AP+MZ regions alone causes MZ properties to be lost; second, treatment of intact embryos (containing a MZ) with IWR-1 result in the loss of MZ markers, suggesting that the MZ is under active maintenance by Wnt signals until the start of gastrulation (primitive streak formation). These findings therefore suggest that Wnt signalling from the AO could be responsible for the initial induction of the MZ during normal development, which occurs at intrauterine stages of development. Consistent with these findings, we find that Wnt ligands (*WNT-3A*, *-5A*, *-5B* and -*8C*) are expressed in the AO and their receptors strongly expressed in the AP. The MZ strongly expresses genes considered to be targets of Wnt signalling including *AXIN-2* and *LEF-1*, confirming earlier studies (Roeser et al., 1999; Skromne and Stern, 2002; Lee et al., 2022b). *WNT8C* is expressed most strongly and as a gradient, highest posteriorly in the AO (Hume and Dodd, 1993; Skromne and Stern, 2001). Here, we find that nuclear localization of β-catenin, indicating where canonical signalling is active, is also graded in the MZ, with highest levels posteriorly, supporting the notion of a gradient.

A large number of studies in anamniote embryos has revealed that, during very early stages of development, canonical Wnt activity is crucial for defining the ‘dorsal’ side of the embryo, thus defining the site where gastrulation will begin and therefore one of the axes of embryonic polarity (Larabell et al., 1997; Miller et al., 1999; Salic et al., 2000; Dorsky et al., 2002; Weaver et al., 2003; Lu et al., 2011). In anamniote embryos, where the zygotic genome remains largely silent for the first ten or so cell divisions, polarized expression is achieved by localization of maternal components like mRNAs or proteins – here, the dorsally-localized determinant is nuclear localization of β-catenin. In *Xenopus*, it is cortical rotation following fertilization that generates the first dorsoventral difference, determining where the nuclear localization will take place (Larabell et al., 1997). The overlap between canonical Wnt activity and the T-box transcription factor VegT and the TGFβ/GDF-signalling component Vg1 defines the location of the future Nieuwkoop centre, responsible for induction of the organizer in adjacent cells (Brannon and Kimelman, 1996; Carnac et al., 1996; Fagotto et al., 1997).

In the chick embryo, an amniote, a region functionally equivalent to the Nieuwkoop centre has been located to the posterior MZ – it can induce the primitive streak, including the organizer, without contributing any cells to the induced structures (Bachvarova et al., 1998), which are the defining hallmarks of the Nieuwkoop centre in amphibians (Nieuwkoop, 1996a,b, 1973; Harland and Gerhart, 1997). Like the amphibian Nieuwkoop centre, the posterior MZ is a region of overlap of expression between a TGFβ/GDF component (*VG1*) (Seleiro et al., 1996; Shah et al., 1997), the T-box transcription factor TBX6 (Knezevic et al., 1997) and high canonical Wnt activity (this study). This is particularly interesting because, unlike anamniotes, where this region of high Wnt activity is defined by maternal localization resulting from cortical rotation before the activation of zygotic gene expression (see above), in amniotes including the chick, the zygotic genome is activated very early, during the first few cleavage divisions. These results indicate that the ‘dorsal’ localization of Wnt activity, by whatever mechanism, is a highly conserved feature of vertebrate development and essential for the establishment of embryo polarity that will position the site of gastrulation (dorsal blastopore in amphibians, primitive streak in amniotes).

Whether the embryos of eutherian mammals possess a region functionally homologous to the AO of the chick, and whether this region also emits signals that activate the Wnt canonical pathway in the neighbouring epiblast, is not yet clear. Before primitive streak formation in the mouse, at embryonic day 3 post-fertilization (E3), Wnt3 is expressed in the proximal visceral endoderm, close to the boundary between extra-embryonic and embryonic regions of the ectoderm where the primitive streak will later arise (Rivera-Perez and Magnuson, 2005). Wnt3 and β-catenin mutants fail to form a primitive streak (Haegel et al., 1995; Liu et al., 1999). Misexpression of Wnt8 (which activates the canonical Wnt pathway) in all epiblast cells, or manipulation of the expression of Dkk1 (Wnt antagonist) or of β-catenin, affect the anterior migration of the anterior visceral endoderm, which the authors interpreted as indicating that the migration of this tissue is directed by graded canonical Wnt-activity along the anterior-posterior axis (Kimura-Yoshida et al., 2005). However, these findings are also consistent with the possibility that this signalling pathway also plays a role more upstream, by defining the posterior end of the epiblast. We hope that our present findings in the chick embryo will stimulate research into this in the mouse as well as in non-rodent mammals. Key questions remaining include: is there a crucial region situated between extra-embryonic ectoderm and the embryonic epiblast that plays a role in embryo polarity but without contributing cells to the primitive streak (like the chick MZ), and is this region also defined and maintained by Wnt activity from more remote extra-embryonic tissues?

Cooperation or synergy between TGFβ and canonical Wnt signalling seems to be even more highly conserved – even in the fly, several different developmental events involve both pathways (Bilder et al., 1998; Estella and Mann, 2008; Requena et al., 2017). In vertebrates, different TGFβ ligands can have opposing functions. While activin/Nodal/GDF3/GDF1 (which activate Smad2/3) act to promote primitive streak formation posteriorly, BMPs (which activate Smad1/5/8) act to inhibit primitive streak formation elsewhere in the embryo. Does canonical Wnt cooperate with both of these opposing activities? An earlier study exploring the role of VG1 in primitive streak formation in the chick reported that when VG1 is misexpressed anywhere in the MZ, it can induce an ectopic primitive streak in adjacent AP epiblast; however, when VG1-expressing cells are placed into the AP itself, no expression of primitive-streak markers is induced (Skromne and Stern, 2001). Because of the conservation of Wnt/TGFβ interactions, the study then explored whether differences in Wnt activity could underlie this difference. Indeed, when cells expressing Wnt1 are co-transplanted with cells expressing VG1 into the AP, a region of TBXT (Brachyury) expression (primitive streak marker) is induced next to the grafted cells (Skromne and Stern, 2001). Moreover, when VG1 is misexpressed in the anterior MZ together with cells secreting Wnt antagonists like NFz8, this no longer induces an ectopic streak (Skromne and Stern, 2001). Although that study was performed with transfected cells as the source of factors (and it is possible that other factors produced by the cells also contribute to the results), the paper proposed that interactions between Vg1 and Wnt are important in the induction of primitive streak formation in early chick development, as shown in *Drosophila* and other systems. Our present study opens a refinement of this interpretation: it is not just Wnt activity that is required for VG1 to induce a primitive streak, but it may be that it is the MZ character induced by Wnt that is important. Thus, Wnt would define the ring of MZ properties all around the margin of the embryo, and the localization of VG1 (and TBX6) would define its posterior part, where the inducing molecules are produced. These speculations are supported by the present findings that incubation of isolated AP fragments in the Wnt agonist BIO generates broad expression of MZ genes (*GJB6* and *ASTL*) and concomitant loss of the AP specific gap-junction marker *GJA1*. Whether canonical Wnt activity also promotes the activity of BMPs in inhibiting primitive streak formation other than next to the posterior MZ remains to be investigated, but the present study, showing that Wnt activity is seen all around the MZ, suggests that it might.

We noted that an endogenous inhibitor of canonical Wnt signalling, *DKK1*, is expressed in a domain overlapping with *WNT8C* and its target *AXIN2* (Arendt and Nübler-Jung, 1999; Foley et al., 2000; Chapman et al., 2002; Skromne and Stern, 2002; Lee et al., 2020). Moreover, Wnt antagonists of the sFRP (secreted frizzled receptor protein) family are expressed in the AP (Skromne and Stern, 2002; Lee et al., 2020), and an additional Wnt modulator, *GPC-4* (glypican-4), which facilitates the diffusion of Wnt and may act as an inhibitor by binding the ligand (Ohkawara et al., 2003) is expressed in the hypoblast, just beneath the epiblast of the AP. These observations suggest that Wnt activity is more delicately controlled and positioned than merely by the localization of a Wnt ligand and its receptors. This is worthy of future investigation.

During normal development, the MZ is established around stages EGK VIII-IX (Eyal-Giladi and Kochav, 1976; Kochav et al., 1980), when the egg is still within the maternal oviduct. Technical limitations make it impossible at present to conduct experiments at these stages. However, the findings of the present study, along with its predecessor (Lee et al., 2022b), make it very likely that Wnt activity from the AO is involved in the initial appearance of the MZ at these early stages, by a process of instructive induction (Gurdon, 1987). The present study reveals that the continued presence of the AO is required to maintain the MZ at later stages, therefore this inductive interaction may be a continuous one. The previous study (Lee et al., 2022b) also revealed that the AO loses its ability to induce MZ properties from AP epiblast when the primitive streak appears at stage HH2. Here, we show that *WNT8C* expression disappears from the AO after primitive streak formation, as does the expression of receptors and targets of the canonical Wnt pathway from the MZ and AP – the components of canonical Wnt signalling move to the primitive streak itself at this stage, suggesting that the developmental functions of this pathway become different as gastrulation begins. Together, our findings suggest that, during normal development, Wnt signals from the AO induce the MZ from AP epiblast, all around the periphery. This interaction persists, and is required continuously, until the primitive streak starts to form at stage HH2.

## MATERIALS AND METHODS

### Embryo harvest and New culture

Fertile Dekalb White hens' eggs (Henry Stewart & Co., UK) were incubated at 38°C for 2 h to obtain embryos at stages EGK X-XI. Embryos were explanted from the egg and manipulated in Pannett-Compton saline (Pannett and Compton, 1924), phosphate buffered saline (PBS) or Tyrode's solution (Tyrode, 1910) as previously described (Stern, 1993; Streit and Stern, 2008). They were then set up for modified New culture as previously described (New, 1955; Stern and Ireland, 1981) and incubated for the desired length of time as indicated in the text. Then they were fixed as required for each procedure described below.

### Pharmacological treatments

The Wnt agonist BIO (B1686; Sigma-Aldrich) and Wnt antagonist IWR-1 (I0161; Sigma-Aldrich) were stored as 5 μl aliquots at 10 mM and 25 mM, respectively, in DMSO at −20°C. Two dilutions were used: 1:500 in PBS for pre-soaking (30 min at room temperature) and 1:1000 for New culture. Treatments were diluted in 1 ml PBS then mixed with thin albumin to make up a final working concentration of 25 μM for IWR-1 and 10 μM for BIO; 0.2% DMSO was used for controls. Incubation in vehicle (0.2% DMSO) alone had no effect: embryos grown for 6 h with 0.2% DMSO/albumin did not differ significantly from embryos grown for the same time with albumin alone (8/8 embryos and 5/5 embryos, respectively, expressed VG1 appropriately, like freshly explanted embryos).

### Embryo manipulations

Whole embryos were cultured for 6 h to reach approximately stages EGK XII-XIII. For MZ removal experiments, the MZ was excised using a bent entomological pin (A1). At these stages, the epiblast of the AP, MZ, and AO is a continuous epithelium, largely one cell thick, forming a disc with an overall diameter of ∼3 mm (Eyal-Giladi and Kochav, 1976; Kochav et al., 1980; Lee et al., 2020, 2024a). The inner boundary between the MZ and AP was evident by the lack of germ wall cells at the AP side and by differences in the opacity/refractivity of the epiblast concomitant with the change in cell shape at that boundary (Bancroft and Bellairs, 1974; Eyal-Giladi and Kochav, 1976). The boundary between the AO and the MZ was identified by the darker cells of the AO and by the attachment of germ wall cells to the overlying epiblast in the latter. Ablation of the AO was performed by scoring along its inner and outer boundaries (but see next sentence) with a mounted entomological pin. A 15° arc from the posterior part of the AO was also ablated. After ablation, the remaining AO was wrapped around the AP to encircle it completely (within the plane), as previously described (Lee et al., 2022b) (see also Fig. 3A,B). To ensure that the tissues to be used for recombinants and ablation experiments were not contaminated with the neighbouring region, we always erred on the side of caution, removing a little more than the anatomical region in ablation experiments, and a little less when obtaining tissue for grafting. To confirm that the anatomical criteria were being applied correctly, we performed *in situ* hybridization for MZ markers in these conditions (Fig. 3B; Fig. S1). The AO (after excision of about 45° arc from the posterior part) was then grafted onto the AP and the two tissues sealed together by aspiration of as much liquid as possible using a fine micro-needle pulled from a 50 μl borosilicate capillary tube (Streit and Stern, 2008; Lee et al., 2022b). Operated embryos were cultured for 7 h to reach stage EGK XIII-XIV. For AP experiments, both the MZ and the AO were removed.

### Fixation and whole-mount *in situ* hybridization

Embryos and embryo conjugates were fixed with 4% paraformaldehyde in calcium/magnesium-free PBS (PFA) containing 2 mM EGTA (pH 7.4) overnight (up to 15 h) at 4°C. The next day, embryos were transferred to 100% methanol and stored for up to 3 days at −20°C before *in situ* hybridization. Whole mount *in situ* hybridization was performed as previously described (Stern, 1998; Streit and Stern, 2001). The probes used were: *cVG1* (Shah et al., 1997), *cBRA/TBXT* (Kispert et al., 1995), *ASTL* (ChEST817d16) (Lee et al., 2020), *GJB6* (ChEST89h10), *GJA1* (kind gift of Prof. Stephen Price, University College London, UK), *WNT8C* (Hume and Dodd, 1993), *LEF-1* (Kengaku et al., 1998) and *TBX6* (Torlopp et al., 2014). Images of the stained embryos were taken using an Olympus SZH10 dissecting microscope with a QImaging Retiga 2000R camera using QCapture Pro software.

### β-catenin immunohistochemistry, confocal imaging and image analysis

Embryos at around stage EGK-XI were fixed in PFA for 1 h, then washed with ice-cold methanol for 30 min. They were then rehydrated in a dilution series of methanol (75/50/25%) and PBS containing 1% Triton-X100 (PBST), then washed three times (15 min per wash) in PBST followed by 2 h blocking in PBST containing 5% normal goat serum and 1% Thimerosal at room temperature. After blocking, embryos were incubated overnight at 4°C in mouse monoclonal antibody against β-catenin (1:200) (C7207; Sigma-Aldrich). The following day embryos were washed for 10 min three times in PBST, followed by three 1 h washes. Goat-anti mouse IgG Alexa-Fluor 594 (A11032; Invitrogen) was used as secondary antibody (1:500) in blocking buffer, with overnight incubation at 4°C. Embryos were then washed in PBST and counterstained for nuclei using 2.5 µg/ml DAPI. Next, embryos were mounted on a glass slide, ventral-side facing up, using Vectashield (H-1000-10) mounting medium and overlain by a glass coverslip. Confocal imaging was carried out using a Leica SPE1 microscope. Images were processed using open-source FIJI (ImageJ) software. Images were obtained by imaging six regions (posterior AO, AP and MZ, and anterior AO, AP and MZ) in three separate embryos. Segmentation was carried out using the DAPI channel of a ten-image *z*-stack, to create a region of interest (ROI) for the nuclei. Then, in the β-catenin channel, the pixel intensity of nuclear β-catenin was measured within the created ROI. A membrane ROI was created, using a ten-image *z*-stack of the β-catenin channel. The intensity of membrane β-catenin was measured using the ‘measure’ function in the ‘analyse particle’ menu. To normalize for differences in cell density and for cell size, we divided the pixel intensity of β-catenin in the membrane by the β-catenin intensity within the ROI of the nuclei.

### Definitions of terms

In the text, we have used the term ‘new’ MZ to mean that, after removal of all endogenous MZ cells, new cells (not previously part of the MZ) now acquire key properties that are unique to the MZ. In our previous paper (Lee et al., 2022b) we showed that the cells that become this new MZ are primarily central (AP) epiblast, therefore we suggested that the AO induces MZ character in the (neighbouring/continuous) layer of epiblast of the AP when the MZ is removed. All this occurs within the same one-cell-thick layer.

We use ‘MZ identity’ when the description is based on molecular markers and cell morphology, ‘functional MZ’ when we have also assessed some of its defining functional properties, and ‘new’ to refer to a combination of molecular and other criteria.

### Graphs and illustrations

We used GraphPad Prism version 9.5.1 (528) for all graphs and statistical analysis. Figs 1A, 2A and 3A were created using BioRender.com (see legends for links). For Fig. S1A the illustrations were adapted from ‘Wnt signalling (active) and Wnt signalling (inactive)’, by BioRender.com (2024), retrieved from https://app.biorender.com/biorender-templates.

## Supplementary Material

10.1242/develop.204350_sup1 Supplementary information

Table S1. Ligand-receptor expression comparisons from CellChatDB analysis.
