# Supplementary material for: Canonical Wnt signalling from the area opaca induces and maintains the marginal zone in pre-primitive-streak stage chick embryos
Source: Development. 2025 Jan 23;152(2):dev204350. doi: 10.1242/dev.204350 (PMC11829775; doi:10.1242/dev.204350)
Supplement: Supplementary information [file develop-152-204350-s1.pdf]

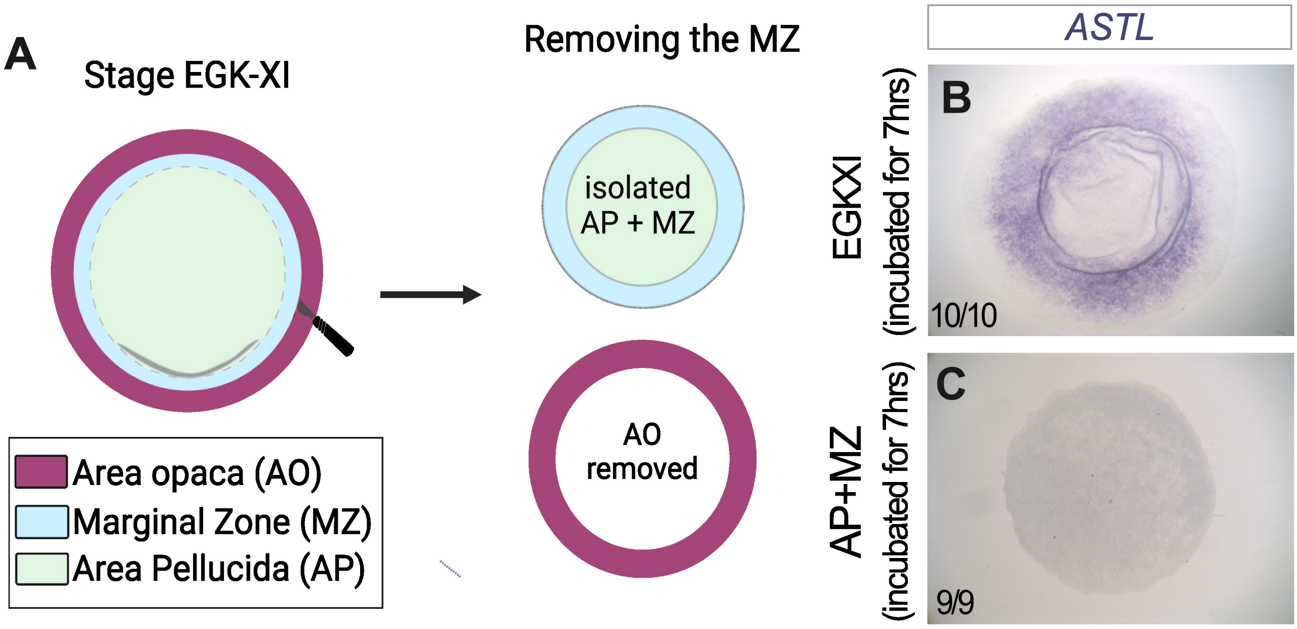

Fig. S1. The MZ is continuously maintained by the AO. A. Schematic diagram of the experimental design. **B-C.** In situ hybridisation for MZ marker *ASTL* in normal, intact EGK-XI embryos (B) or EGK-XI embryos from which the AO had been removed and the MZ+AP fragment incubated (C).

Table S1. Ligand-receptor expression comparisons from CellChatDB analysis.

Available for download at
https://journals.biologists.com/dev/article-lookup/doi/10.1242/dev.204350#supplementary-data

Development • Supplementary information
